# Identification of porcine PARP11 as a restricted factor for pseudorabies virus

**DOI:** 10.3389/fcimb.2024.1414827

**Published:** 2024-10-09

**Authors:** Chunyun Qi, Dehua Zhao, Xi Wang, Lanxin Hu, Yao Wang, Heyong Wu, Feng Li, Jian Zhou, Tianyi Zhang, Aosi Qi, Yuran Huo, Qiuse Tu, Shuyu Zhong, Hongming Yuan, Dongmei Lv, Shouqing Yan, Hongsheng Ouyang, Daxin Pang, Zicong Xie

**Affiliations:** ^1^ Key Laboratory of Zoonosis Research, Ministry of Education, College of Animal Sciences, Jilin University, Changchun, Jilin, China; ^2^ Chongqing Research Institute, Jilin University, Chongqing, China; ^3^ Center for Animal Science and Technology Research, Chongqing Jitang Biotechnology Research Institute Co., Ltd, Chongqing, China; ^4^ Laboratory of Biotechnology and Biomedical Research, Shenzhen Kingsino Technology Co., Ltd., Shenzhen, China

**Keywords:** PARP11, PRV, mRNA export, autophagy, host factor

## Abstract

**Introduction:**

PRV infection in swine can cause devastating disease and pose a potential threat to humans. Advancing the interplay between PRV and host is essential to elucidate the pathogenic mechanism of PRV and identify novel anti-PRV targets.

**Methods:**

PARP11-KO PK-15 cells were firstly constructed by CRISPR/Cas9 technology. Next, the effect of PARP11-KO on PRV infection was determined by RT-qPCR, TCID50 assay, RNA-seq, and western blot.

**Results and discussion:**

In this study, we identified PARP11 as a host factor that can significantly affect PRV infection. Inhibition of PARP11 and knockout of PARP11 can significantly promoted PRV infection. Subsequently, we further found that PARP11 knockout upregulated the transcription of NXF1 and CRM1, resulting in enhanced transcription of viral genes. Furthermore, we also found that PARP11 knockout could activate the autophagy pathway and suppress the mTOR pathway during PRV infection. These findings could provide insight into the mechanism in which PARP11 participated during PRV infection and offer a potential target to develop anti-PRV therapies.

## 1 Introduction

Pseudorabies virus (PRV), the causative agent of pseudorabies, is an enveloped double-stranded DNA (dsDNA) virus that belongs to the subfamily Alphaherpesvirinae (Zhang and Tang, 2021). PRV infection in swine can result in devastating disease and huge economic losses in the swine industry. Although pigs are the natural host of PRV, the virus can also infect numerous other species such as ruminants and rodents (Woźniakowski and Samorek-Salamonowicz, 2015; Wong et al., 2019). Especially, accumulating evidence have shown that variant PRV can directly infect humans, which leads to severe damage in nervous and respiratory systems (Liu et al., 2021; Wong et al., 2019). These findings indicate that PRV infection is not only a threat to the swine industry but also a potential public health risk. Therefore, identifying effective antiviral targets against PRV to prevent and control PRV infection is essential.

ADP-ribosylation is a highly conserved, fundamental post-translational modifications (PTMs) that impacts the regulation and maintenance of many cellular processes, including intracellular innate immune response (Boehi et al., 2021; Dasovich and Leung, 2023). Recently, accumulating evidence suggested that poly-adenosine diphosphate-ribose polymerases (PARPs) also can be involved in virus infection. PARP9 could inhibit vesicular stomatitis virus (VSV) and reovirus infection in mouse (Xing et al., 2021a). It is reported that PARP11 knockout could inhibit VSV and Sendai virus (SeV) replication via enhancement of type I interferon (IFN-I)-induced signal transducer and activator of transcription 1 (STAT1) activation (Guo et al., 2019). Also, PARP11 can suppress Zika virus (ZIKV) replication in cooperation with PARP12 (Li et al., 2021b). These findings indicate that PARP11 may play different roles in viral infection. However, the involvement of PARP11 in PRV infection is still unknown.

In this study, we investigated the role of PARP11 in PRV infection, and we demonstrated that treatment of PARP11 inhibitor or PARP11 knockout in PK15 cells significantly increased PRV infection. Subsequently, we further found that PARP11 knockout upregulated the expression of nuclear export factor 1 (NXF1) and chromosome region maintenance 1 (CRM1), resulting in enhanced transcription of viral genes. Furthermore, we also found that PARP11 knockout could activate the autophagy pathway and suppress the mTOR pathway during PRV infection. These findings could offer a potential target to develop anti-PRV therapies.

## 2 Materials and methods

### 2.1 Cells and viruses

PK-15 (CCL-33, ATCC), 3D4/21 (CRL-2843, ATCC), HEK293T (CRL-11268, ATCC), Vero (CL-81, ATCC), Marc145, LLC-PK1, and IPEC-J2 were cultured in DMEM (10566016, Gibco) supplemented with 10% fetal bovine serum (FBS), 100 units/ml penicillin, 1% Non-Essential Amino Acids (NEAA, Gibco), and 2 mM L-Glutamine (Gibco). LLC-PK1 and IPEC-J2 were kindly provided by Prof. Bin Li of Institute of Veterinary Medicine, Jiangsu Academy of Agricultural Sciences.

All experiments about viruses were conducted in BSL II laboratory. PRV BarthaK61 strain and PRV field strain were used and preserved at -80°C. The PRV-EGFP recombinant strain was generated according to methods described previously with minor modification. First, two gRNAs targeting the PRV TK gene were synthesized (Comate Bioscience, Changchun, China) and ligated to pX330 plasmid. Second, a donor plasmid for homologous recombination was created using fused PCR. It contained the left homologous region (~650 bp upstream of TK gene), the EGFP expression cassette which was control By CMV promotor, and the right homologous region (800 bp downstream of TK gene). Two sgRNAs and donor plasmid were coelectro-transfected into PK15 cells, and the cells were infected with 5000 50% tissue culture infective dose (TCID_50_) at 24 hours post-transfection. Finally, the EGFP plaques were identified, and the PRV-EGFP was purified to homogeneity following a series of 7–10 rounds of screening and subsequent limited dilution.

### 2.2 Antibodies and chemical reagents

The antibody anti-PARP11 (16692-1-AP) was from Proteintech; anti-LC3 was from Cell Signaling Technology; anti-ACTB (AA128), anti-GAPDH (AG019) were from Beyotime; HRP Conjugated AffiniPure Goat Anti-Rabbit IgG (H+L) (BA1055) was from BOSTER; HRP-labeled Goat Anti-Mouse IgG(H+L) (A0216) was from Beyotime. ITK7 (HY-125218) were from MedChemExpress.

### 2.3 Electroporation and generation of knock out cell clones

Approximately 30 μg pX330 plasmids containing crRNAs targeting PARP11 gene were electro-transfected into ~3×10^6^ PK-15 cells resuspended in 300 μL Opti-MEM (Gibco) in 2 mm gap cuvettes using BTX-ECM 2001. The parameters were as follows: 300 voltage, 1 ms, 3 pulses, 1 repeat.

The PK-15 cells were seeded into ten 100-mm dishes after 48 hours post-transfection, and the inoculation density per dish was 2000 cells on average. The cell clones were picked and continually cultured in 24-well plates. Forty percent of one well were digested for 2 min at 37°C and lysed with 10 μL NP-40 lysis buffer (10 mM Tris-HCl pH 8.3, 50 mM KCl, 1.5 mM MgCl_2_, 1% NP-40, and 1% protease K) for 1 h at 56°C and 10 min at 95°C after each clone reaching into 80% confluency. The lysate was used as PCR template and subjected to Sanger sequencing. The positive PK-15 clones were propagated into 100-mm dishes one step at a time.

### 2.4 Cell viability assay

Cell viability was determined by using Cell Counting Kit-8 (AR1160, BOSTER) according to the manufacturer’s instructions. The absorbance was measured with TECAN Infinite 200 PRO.

### 2.5 Virus titration

Vero cells were seeded in a 96-well plate at 10^4^ per well prior to infection. The cells were inoculated with serially diluted viruses (10^−2^–10^−10^ fold) for 1 h at 37°C. The excess virus inoculum was removed by washing with PBS. Then, 200 μL maintenance medium (DMEM with 2% FBS) was added to each well. The plate was incubated at 37°C for a further 3 to 5 days, followed by observation of the cytopathic effect of each well under a light microscope. The TCID_50_ values were calculated by the Reed–Muench method.

### 2.6 Western blot

Cells were washed in ice-cold phosphate-buffered saline (PBS) and lysed in Cell Lysis Buffer for Western and IP (P0013, BEYOTIME) containing 1mM PMSF (AR1192, BOSTER) and 1% Protease Inhibitor Cocktail (AR1182, BOSTER). The protein concentrations were measured with the BCA assay Kit (AR1189, BOSTER) and 40 μg proteins were diluted in 5×SDS-PAGE Loading Buffer (AR1112, BOSTER) at 95°C for 10 min. Subsequently, the boiled samples were subjected to 10% SDS-PAGE and then transferred to 0.22 μm polyvinylidene difluoride (PVDF) membranes (BOSTER). Membranes were blocked in 5% nonfat dry milk dissolved in TBST for 2 h at room temperature, and then probed with the primary antibodies, followed by the respective HRP-conjugated goat anti-mouse or anti-rabbit secondary antibodies. The following antibodies were used: mouse anti-GAPDH monoclonal antibody (1:1000, AG019 BEYOTIME), mouse anti-ACTB monoclonal antibody (1:1000, AA128 BEYOTIME), PARP11 Polyclonal antibody (1:500, 16692-1-AP, Proteintech), HRP Conjugated AffiniPure Goat Anti-Rabbit IgG (H+L) (BA1055, BOSTER), HRP-labeled Goat Anti-Mouse IgG(H+L) (A0216, Beyotime). Ultimately, membranes were imaged with the ultra-sensitive ECL chemical luminescence ready-to-use kit (AR1197, BOSTER) using Azure c600 (AZUREBIOSYSTEMS). Fiji was used for densitometric analysis of the western blot.

### 2.7 Immunofluorescence assay

Cells grown on 24-well plates were fixed with 4% paraformaldehyde (AR1068, BOSTER) for 30 min at room temperature, and then permeabilized in 0.1% Triton X-100 (T8200, Solarbio) for 10 min at room temperature. The fixed cells were incubated with PBS containing 10% FBS (10% FBS-PBS) with the primary antibody for 1 h at 37°C. After washed with PBS containing 0.05% Tween-20 (T8220, Solarbio), the cells were incubated with fluorescent secondary antibody in a dark, humidified chamber for 1 h at 37°C. Finally, the cells were stained by 4’,6-diamidino-2-phenylindole (DAPI) (C1002, Beyotime). Images were captured on EVOS f1 fluorescence microscope.

### 2.8 Binding, entry, and replication assay

For binding assay, approximately 2×10^5^ cells were infected with PRV at a multiplicity of infection (MOI) of 0.1 in 12-well plates at 4°C for 1 h. The cells were washed thrice with pre-cold PBS and cultured in maintenance medium containing 2% FBS for 24 h. The cells and supernatants were collected to measure virus titers through TCID_50_ assay.

For entry assay, approximately 2×10^5^ cells were infected with PRV (MOI = 0.1) in 12-well plates at 4°C for 1 h. The cells were washed thrice with pre-cold PBS and cultured in complete medium containing 10% FBS for 1 h. Then, to remove the uninternalized viruses, the cells were inoculated with citric acid buffer (pH = 3.0) for 5 min at room temperature. After washed thrice with pre-cold PBS, the cells were continually cultured in maintenance medium containing 2% FBS for 24 h. The cells and supernatants were collected to measure virus titers through TCID_50_ assay.

For replication assay, approximately 2×10^5^ cells were infected with PRV (MOI = 0.1) in 12-well plates at 4°C for 1 h. The cells were washed thrice with pre-cold PBS and cultured in complete medium containing 10% FBS for 12 h. After washed thrice with pre-cold PBS, the cells were continually cultured in maintenance medium containing 2% FBS for 12 h. The cells and supernatants were collected to measure virus titers through TCID_50_ assay.

### 2.9 Real-time quantitative PCR

Total RNA was isolated with RNAsimple Total RNA Kit (DP419, TIANGEN). Cell fractionation was performed using Cytoplasmic & Nuclear RNA Purification Kit (21000, NORGEN). The RNA was subjected to cDNA synthesis with FastKing gDNA Dispelling RT SuperMix (KR118, TIANGEN). RT-qPCR was performed in triplicate using Talent qPCR PreMix (SYBR Green) (FP209, TIANGEN) according to the manufacturer’s instructions and data were normalized to the level of GAPDH expression in each individual sample using the 2^-ΔΔCt^ method. Melting curve analysis indicated formation of a single product in all cases. Primers are shown in **Supplementary Table S1**.

### 2.10 Flow cytometry

Cells were infected with PRV-GFP (MOI = 2). At indicated times, adherent cells were washed once with PBS and then detached by tryptone. Around 1×10^6^ cells were collected into a 1.5-mL centrifuge tube and washed twice with PBS. The pellet was resuspended in 500 μL PBS. Flow cytometry, performed on a CytoFLEX S instrument (BECMAN), was then used to record the GFP intensity per 1×10^4^ cells acquired. Data analysis was performed using CytExpert V2.4.0.28.

### 2.11 Library preparation for transcriptome sequencing

WT and PARP11-KO cells were infected with PRV (MOI = 0.01) for 12 hours post-infection (hpi). Total RNA was isolated with RNAsimple Total RNA Kit (DP419, TIANGEN) and amount of 1 μg RNA per sample was used as input material for the RNA sample preparations. Sequencing libraries were generated using Hieff NGS Ultima Dual-mode mRNA Library Prep Kit for Illumina (Yeasen Biotechnology (Shanghai) Co., Ltd.) following manufacturer’s recommendations and index codes were added to attribute sequences to each sample. Briefly, mRNA was purified from total RNA using poly-T oligo-attached magnetic beads. First strand cDNA was synthesized and second strand cDNA synthesis was subsequently performed. Remaining overhangs were converted into blunt ends via exonuclease/polymerase activities. After adenylation of 3’ ends of DNA fragments, NEBNext Adaptor with hairpin loop structure were ligated to prepare for hybridization. The library fragments were purified with AMPure XP system (Beckman Coulter, Beverly, USA). Then 3 μL USER Enzyme (NEB, USA) was used with size-selected, adaptor-ligated cDNA at 37°C for 15 min followed by 5 min at 95°C before PCR. Then PCR was performed with Phusion High-Fidelity DNA polymerase, Universal PCR primers and Index (X) Primer. At last, PCR products were purified (AMPure XP system) and library quality was assessed on the AgilentBioanalyzer2100 system.

### 2.12 Sequencing and mapping to the reference genome

The above libraries were sequenced on an Illumina NovaSeq platform to generate 150 bp paired-end reads, according to the manufacturer’s instructions. The raw reads were further processed with a bioinformatic pipeline tool, BMKCloud (www.biocloud.net) online platform. Clean data were obtained by removing reads containing adapter, reads containing ploy-N and low-quality reads from raw data. These clean data were then mapped to the reference genome sequence (https://ftp.ensembl.org/pub/release-109/fasta/sus_scrofa/dna/Sus_scrofa.Sscrofa11.1.dna.toplevel.fa.gz). Only reads with a perfect match or one mismatch were further analyzed and annotated based on the reference genome. Hisat2 tools were used to map with reference genome.

### 2.13 Differential expression analysis and enrichment analysis

Differential expression analysis of PARP11-KO/WT was performed using the DESeq2. DESeq2 provide statistical routines for determining differential expression in digital gene expression data using a model based on the negative binomial distribution. The resulting *P* values were adjusted using the Benjamini and Hochberg’s approach for controlling the false discovery rate. Genes with an adjusted *P*-value < 0.01 & Fold Change≥2 found by DESeq2 were assigned as differentially expressed. The clusterProfiler software to test the statistical enrichment of differential expression genes in KEGG pathways.

### 2.14 Statistical analysis

Statistical analysis was performed using GraphPad Prism 8.0 software. Unpaired Student’s *t*-tests were used to compare two groups. The significance levels are *P < 0.05, **P < 0.01, ***P < 0.001 and ****P < 0.0001.

## 3 Results

### 3.1 PRV infection reduces the endogenous expression of PARP11

**Figure 1:**
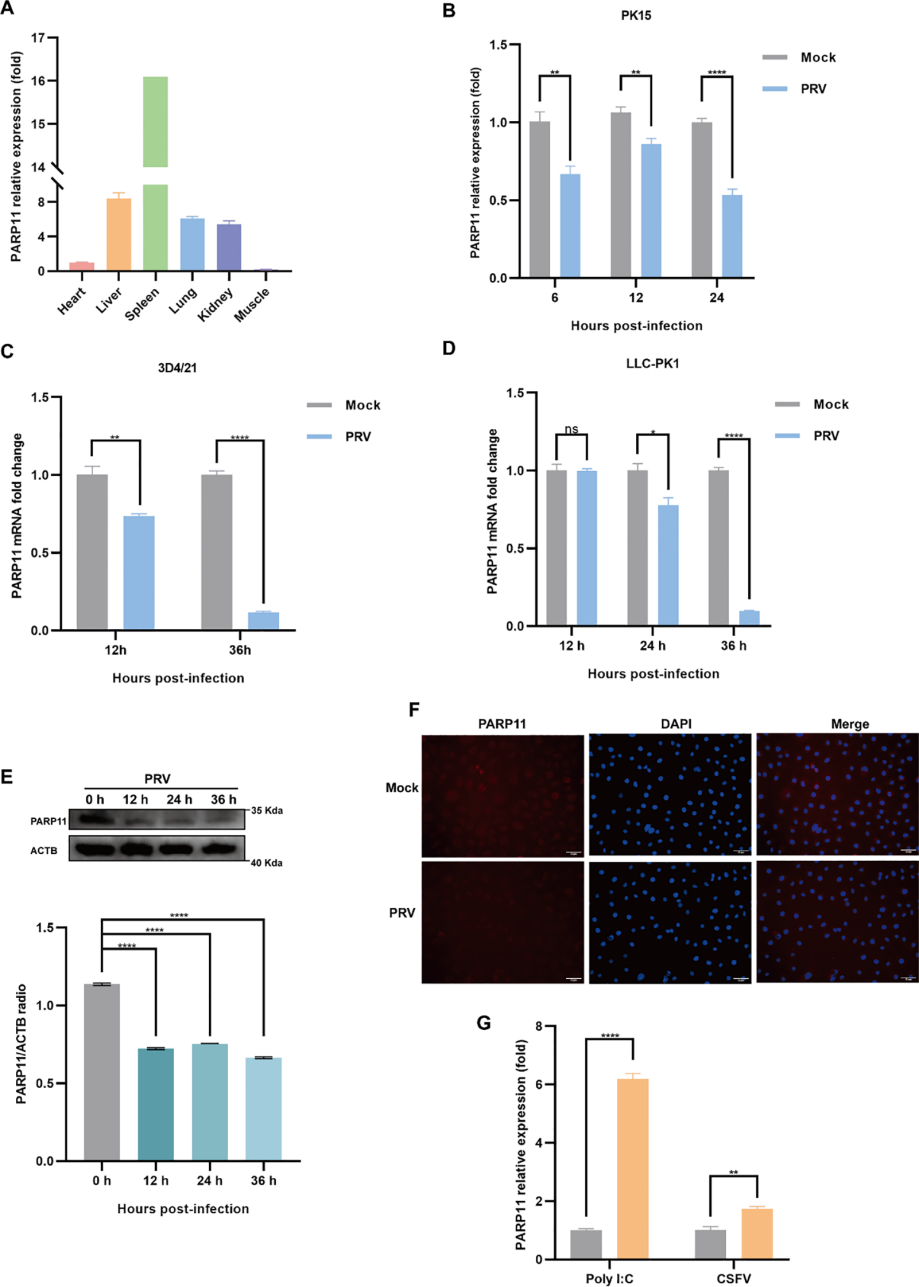
PRV infection decreased the expression of PARP11. **(A)** The mRNA levels of PARP11 in various organs from Large White piglet was determined by RT-qPCR. The mRNA level of PARP11 in heart was used as standard level. The organs were obtained from our previous report (Xie et al., 2018). PK15 **(B)**, 3D4/21 **(C)**, and LLC-PK1 **(D)** were infected with PRV (MOI = 0.01) for the indicated times. PARP11 mRNA level was assessed by RT-qPCR analysis. **(E)** Western blot analysis of PARP11 protein levels in PK-15 cells infected with PRV (MOI = 0.01) for 12, 24, and 36 h. ACTB serves as a loading control and PARP11 abundance was quantified using Fiji software. **(F)** Immunofluorescence analysis of PARP11 protein levels in Marc145 cells infected with PRV (MOI = 0.01) for 24 h. **(G)** PK15 cells were transfected with poly (I:C) for 24 h or infected with CSFV shimen (MOI = 0.1) for 48 h. PARP11 mRNA level was assessed by RT-qPCR analysis. Data are presented as means ± SE of three independent experiments, and asterisks (*) indicate the statistical significance: ns, no significance, *P < 0.05, **P < 0.01, and ****P < 0.0001.

To examine the effects on PARP11 following PRV infection, we determined the mRNA and protein levels of PARP11 in PRV-infected cells. The relative expression of PARP11 in various organs was first elucidated (**Figure 1A**), and we found that the mRNA levels of PARP11 were significantly reduced in PK15, 3D4/21, LLC-PK1 (**Figures 1B–D**) and IPEC-J2 cells (**Supplementary Figure S1A**) during PRV infection. Next, the protein levels of PARP11 were also determined following PRV infection. The western blot analysis indicated that the protein levels of PARP11 were also reduced in PRV-infected PK-15 (**Figure 1E**) and Marc145 cells (**Supplementary Figure S1B**). Immunofluorescence assay also suggested that PRV infection downregulated PARP11 (**Figure 1F**). However, the stimulation of the RNA virus analogue poly(I:C) and classical swine fever virus (CSFV) infection could upregulate the mRNA levels of PARP11 (**Figure 1G**). These results revealed that PRV infection downregulated PARP11 expression in different cell lines.

### 3.2 PARP11 inhibitor and knockdown of PARP11 can promote PRV proliferation

**Figure 2:**
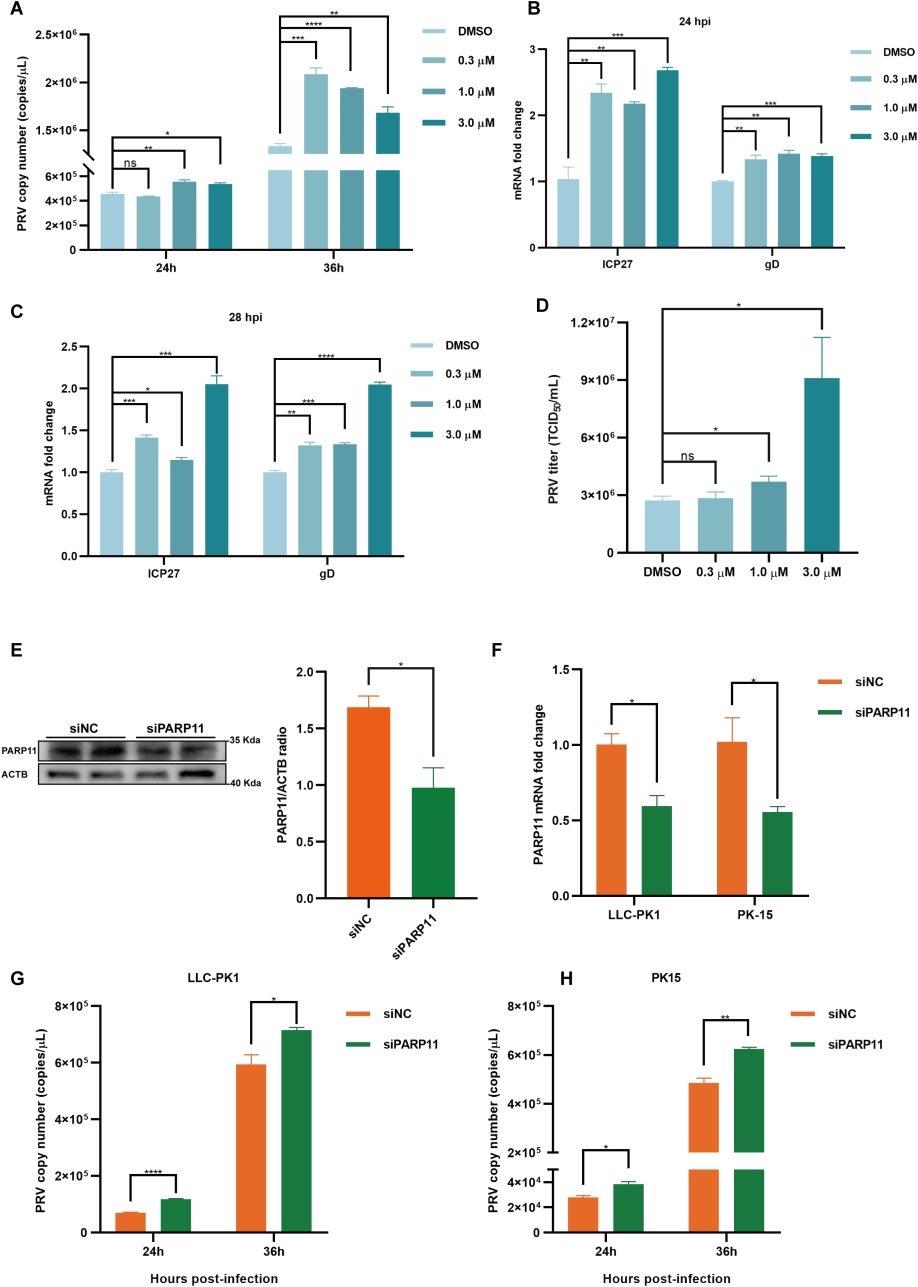
PARP11 inhibitor ITK7 promoted PRV infection. **(A)** PRV genome copies in PK15 cells infected with PRV (MOI = 0.01) and simultaneously treated with ITK7 (0.3–3 μM) for 24, 28 h. The relative expression of PRV ICP27 and gD in ITK7-treated PK15 cells at 24 hpi **(B)** and 28 hpi **(C)**. PK15 cells were infected with PRV (MOI = 0.01) and simultaneously treated with ITK7 (0.3–3 μM) for 24 and 28 h. PRV ICP27 and gD mRNA level was assessed by RT-qPCR analysis. **(D)** PRV titers in PK15 cells infected with PRV field strain (MOI = 0.01) and simultaneously treated with ITK7 (0.3–3 μM) for 24 h. PRV progeny titers were detected by TCID_50_ assay. **(E)** Western blot analysis of PARP11 protein levels in PK-15 cells transfected with siPARP11. PK-15 cells were transfected with scrambled siRNA (siNC) or siRNAs targeting PARP11 for 24 hours. ACTB serves as a loading control and PARP11 abundance was quantified using Fiji software. **(F)** LLC-PK1 and PK15 cells were electro-transfected with siPARP11 for 36 h. PARP11 mRNA level was assessed by RT-qPCR analysis. LLC-PK1 **(G)** and PK15 **(H)** cells were electro-transfected with siPARP11 for 12 h. and then infected with PRV (MOI = 0.01) for 24 and 36 h. PRV genome copies at indicated time were detected. Data are presented as means ± SE of three independent experiments, and asterisks (*) indicate the statistical significance: ns, no significance, *P < 0.05, **P < 0.01, ***P < 0.001, and ****P < 0.0001.

We first analyzed the effects of PARP11 inhibitors on cell viability. HEK293T and PK15 cells were treated with the PARP1 inhibitors ITK7 (0.3~3 μM). Cell viability was determined with Cell Counting Kit-8 (CCK-8) assays. As shown in **Supplementary Figures S1C, D**, PARP11 inhibitors ITK7 were harmless to both types of cells. Next, we analyzed the effect of ITK7 on PRV infection. PARP11 inhibitors ITK7 significantly promoted PRV infection in PK15 cells (**Figure 2A**). The mRNA level of PRV early gene ICP27 and late gene gD were universally increased at 24 and 28 hpi (**Figures 2B, C**). Similarly, the mRNA level of PRV early gene ICP27 was also increased in ITK7-treated HEK293T cells (**Supplementary Figure S1E**). Furthermore, the PRV progeny titers were also detected by TCID_50_ assay. As shown in **Figure 2D**, the titers of progeny virus increased following ITK7 treatment.

To further determine whether infection of PRV was negative correlation with PARP11 expression, we knocked down endogenous PARP11 by transfecting PK-15 and LLC-PK1 cells with synthesized PARP11-specific siRNA (siPARP11) for 24 h. The results showed that the protein level (**Figure 2E**) and mRNA level (**Figure 2F**) of PARP11 in siPARP11-transfected cells significantly dropped compared with that in siNC-transfected cells. Similar to the effect of ITK7 on PRV infection, viral genome copies assay indicated that knockdown of PARP11 promoted PRV infection (**Figures 2G, H**). The mRNA levels of PRV ICP27 were notably increased in siPARP11-transfected cells (**Supplementary Figure S1F**). These data indicated that inhibition of PARP11 promoted PRV proliferation.

### 3.3 PARP11 knockout promotes PRV proliferation

**Figure 3:**
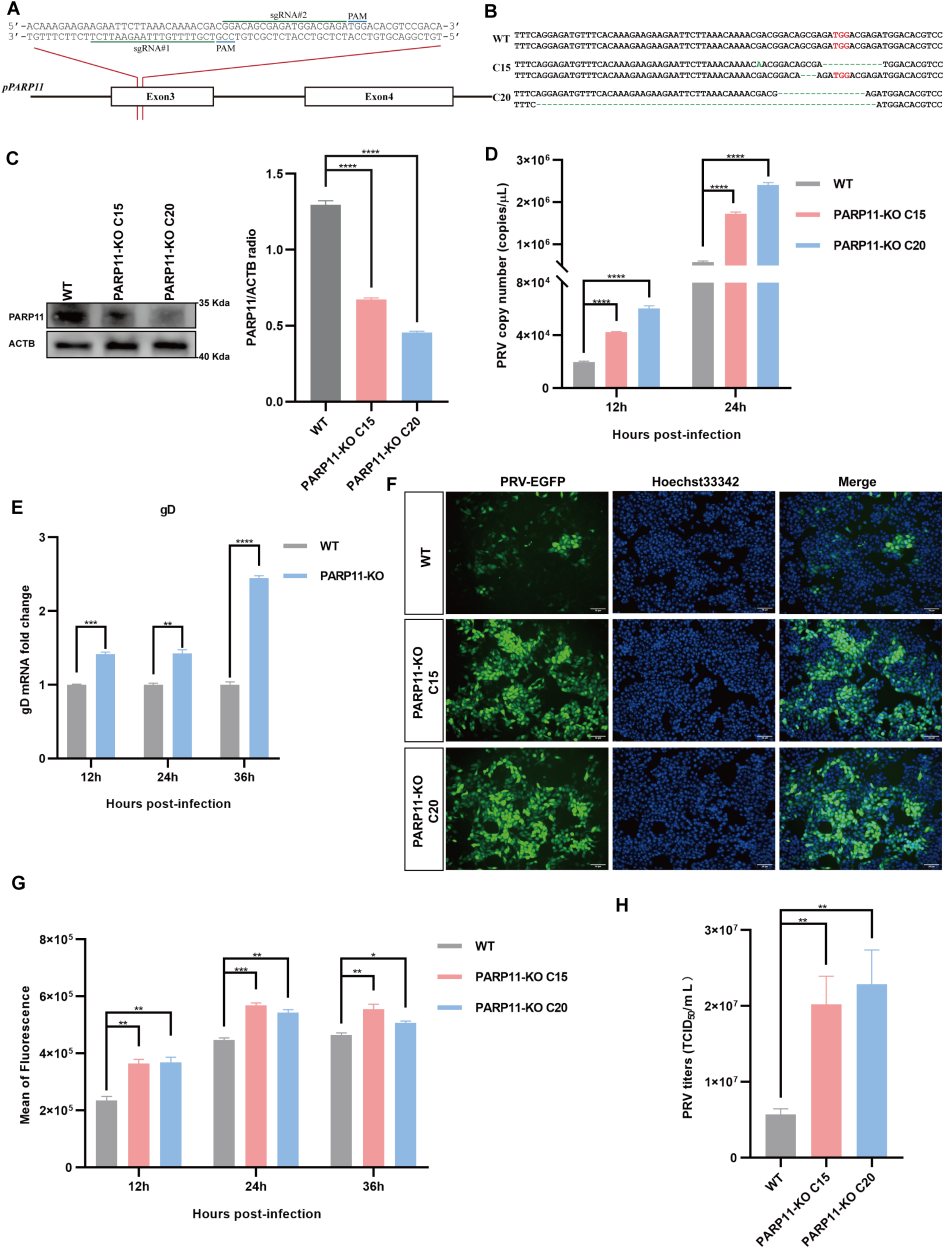
Knockout of PARP11 promotes PRV infection. **(A)** A targeting diagram of representative sgRNAs on the PARP11 locus. **(B)** T-cloning and Sanger sequencing of PARP11-KO PK15 clones. The targeting region on PARP11 locus was amplified and then the amplicons were ligated into T vectors. PAM sites are highlighted in red. **(C)** Western blot analysis of PARP11 protein levels in PARP11-KO cells. ACTB serves as a loading control and PARP11 abundance was quantified using Fiji software. **(D)** PRV genome copies in PARP11-KO cells. WT and PARP11-KO cells were infected with PRV (MOI = 0.01) for 12, 24, and 36 h. PRV genome copies were detected by qPCR analysis. **(E)** PRV gD relative expression in PARP11-KO cells. WT and PARP11-KO cells were infected with PRV (MOI = 0.01) for 12, 24, and 36 h. Total RNA was extracted to detect the mRNA level of PRV gD. **(F)** WT and PARP11-KO cells were infected with PRV-GFP (MOI = 0.01) for 24 h. The fluorescence of GFP was detected by fluorescent microscopy. **(G)** WT and PARP11-KO cells were infected with PRV-GFP (MOI = 2) for 12, 24, and 36 h. The GFP intensity of PRV-EGFP-infected cells was quantified by flow cytometry. **(H)** PRV titers in PARP11-KO cells. WT and PARP11-KO cells were infected with PRV (MOI = 0.01) for 24 h. The supernatant was assessed by TCID_50_ assay. Data are presented as means ± SE of three independent experiments, and asterisks (*) indicate the statistical significance: ns, no significance, *P < 0.05, **P < 0.01, ***P < 0.001, and ****P < 0.0001.

To further confirm that inhibition of PARP11 enhanced PRV infection, we knocked out PARP11 expression in PK15 cells by CRISPR/Cas9. Two specific sgRNAs targeting porcine PARP11 exon 3 were designed (**Figure 3A**). Two PARP11 knockout (PARP11-KO) PK15 clones were picked by limited dilution and sanger sequencing confirmed that the PARP11 sequence had been disrupted successfully (**Figure 3B**). Western blot assay showed that PARP11 protein levels in knockout cells were significantly reduced (**Figure 3C**), and CCK-8 cell counting assay indicated that PARP11-KO did not result in a limitation of cell proliferation (**Supplementary Figure S2A**).

Subsequently, to determine the role of PARP11-KO in the proliferation efficiency of PRV, we infected WT and PARP11-KO cells with PRV. The effect of PARP11-KO on PRV genome copy number was examined by quantitative real-time PCR (RT-qPCR). As shown in **Figure 3D**, the PRV genome copies in PARP11-KO cells were higher than that in WT cells. The mRNA levels of gD (late gene) were increased in PARP11-KO cells (**Figure 3E**). Besides, we also detected the GFP intensity in recombinant PRV-EGFP-infected cells. It was found that PARP11 knockout cells had a significantly higher EGFP intensity than WT cells, suggesting that PARP11 knockout promoted PRV-EGFP infection (**Figures 3F, G**). The TCID_50_ assay also showed higher titers in PARP11-KO cells (**Figure 3H**, **Supplementary Figure S2B**). These results demonstrated that knockout of PARP11 promoted PRV infection.

### 3.4 PARP11 knockout affects PRV replication phase during the infection

**Figure 4:**
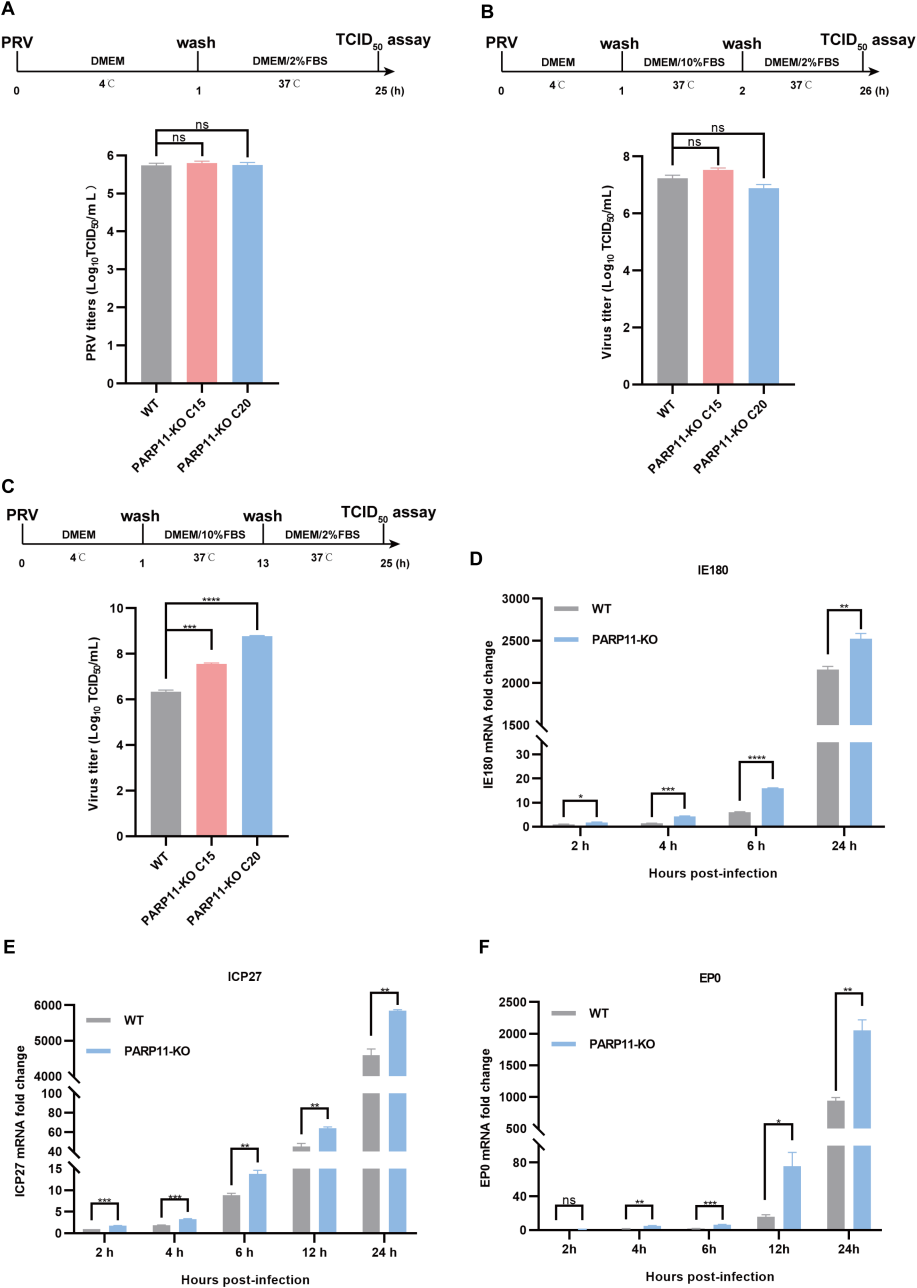
PARP11 knockout affects PRV replication. **(A)** WT and PARP11-KO cells were incubated with PRV (MOI = 0.1) for 1 h at 4°C. After washing with cold PBS three times, cells were continuously cultured in DMEM with 2% FBS for 24 h at 37°C. The supernatant and cell pellet were freeze-thawed and subjected to a TCID_50_ assay to determine PRV titers. **(B)** WT and PARP11-KO cells were incubated with PRV (MOI = 0.1) for 1 h at 4°C and then cultured in DMEM with 10% FBS for 1 h at 37°C. After 1 h to allow viral entry, cells were cultured in DMEM with 2% FBS for 24 h at 37°C. The supernatant and cell pellet were freeze-thawed and subjected to a TCID_50_ assay to determine PRV titers. **(C)** WT and PARP11-KO cells were incubated with PRV (MOI = 0.1) for 1 h at 4°C. After washing with cold PBS three times, cells were cultured in DMEM with 10% FBS for 12 h at 37°C. Cells were then cultured in DMEM with 2% FBS for 12 h at 37°C. The supernatant and cell pellet were freeze-thawed and subjected to a TCID_50_ assay to determine PRV titers. **(D–F)** WT and PARP11-KO cells were infected with PRV (MOI = 0.1) for 2, 4, 6, 12, and 24 h. The mRNA levels of PRV IE180 **(E)**, ICP27 **(F)** and EP0 **(G)** were assessed by RT-qPCR analysis. Data are presented as means ± SE of three independent experiments, and asterisks (*) indicate the statistical significance: ns, no significance, *P < 0.05, **P < 0.01, ***P < 0.001, and ****P < 0.0001.

Based on findings that PARP11-KO can enhance PRV proliferation, we further investigated the effects of PARP11 on PRV attachment, internalization, and replication. Compared with WT cells, there were no significant differences in PRV attachment and internalization in PARP11-KO PK15 cells (**Figures 4A, B**). Notably, in the replication phase, the multiplication of PRV was increased significantly in PARP11-KO PK15 cells than in WT PK15 cells (**Figure 4C**). Furthermore, the mRNA levels of PRV IE180 (immediate-early gene) and ICP27 (early gene) were increased as early as 2 h post PRV infection (**Figures 4D, E**). The mRNA levels of PRV EP0 (early gene) were increased starting at 4 h post infection (**Figure 4F**). These data showed that PARP11 can participate in the replication process during PRV proliferation.

### 3.5 PARP11 knockout promotes the transcription of mRNA processing and mRNA export pathway

**Figure 5:**
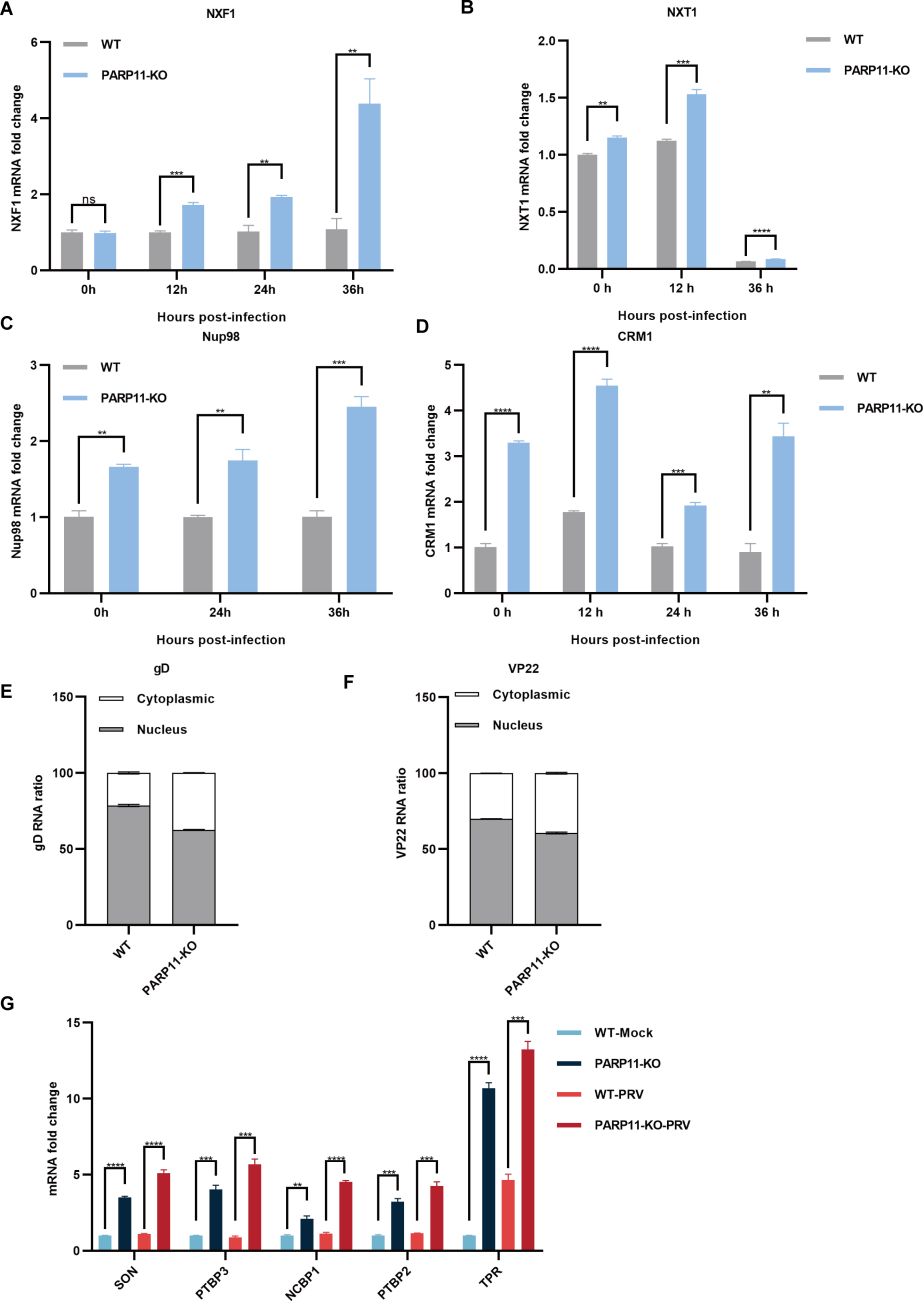
PARP11 knockout facilitated RNA processing and mRNA export. **(A–D)** The representative genes expression of mRNA export in PARP11-KO cells. WT and PARP11-KO cells were infected with PRV (MOI = 0.01) for 0, 12, 24, and 36 h. NXF1 **(A)**, NXT1 **(B)**, Nup98 **(C)**, and CRM1 **(D)** were detected by RT-qPCR analysis. The distribution of cytoplasmic and nuclear PRV gD **(E)** and VP22 **(F)** mRNAs in PARP11-KO cells at 24 hpt was analyzed using RT-qPCR. **(G)** The representative genes expression of RNA processing in PARP11-KO cells. WT and PARP11-KO cells were infected with PRV (MOI = 0.01) for 0 and 12 h. SON, PTBP2, PTBP3, NCBP1, and TPR were detected by RT-qPCR analysis. Data are presented as means ± SE of three independent experiments, and asterisks (*) indicate the statistical significance: ns, no significance, **P < 0.01, ***P < 0.001, and ****P < 0.0001.

Previous reports revealed that the mRNA export pathways were usually hijacked by viruses to promote viral replication (Chutiwitoonchai and Aida, 2016; Wendt et al., 2020, 2022; Zhang et al., 2022). To determine whether PARP11-KO promoted PRV replication by affecting mRNA export, we detected the transcription of NXF1, nuclear transport factor 2 like export factor 1 (NXT1), CRM1 as well as nucleoporin 98 and 96 precursor (Nup98) in PARP11-KO cells following PRV infection. As shown in **Figures 5A–D**, the mRNA levels of NXF1, NXT1, CRM1 and Nup98 were significantly increased in PARP11-KO cells. Accordingly, the mRNA cytoplasmic distribution of PRV gD and VP22 in PARP11-KO cells was higher than in WT cells (**Figures 5E, F**). In terms of the results above, we speculated that the RNA processing pathway might also be involved in the PRV enhancement caused by PARP11-KO. As expected, the transcription levels of RNA processing factors including SON DNA and RNA binding protein (SON), polypyrimidine tract binding protein 2 (PTBP2), polypyrimidine tract binding protein 3 (PTBP3), nuclear cap binding protein subunit 1 (NCBP1), and translocated promoter region, nuclear basket protein (TPR) were universally upregulated in PARP11-KO cell (**Figure 5G**). These data suggested that PARP11-KO might activate RNA processing and RNA export pathway during PRV infection.

### 3.6 PARP11 knockout activates autophagy pathway during PRV infection

**Figure 6:**
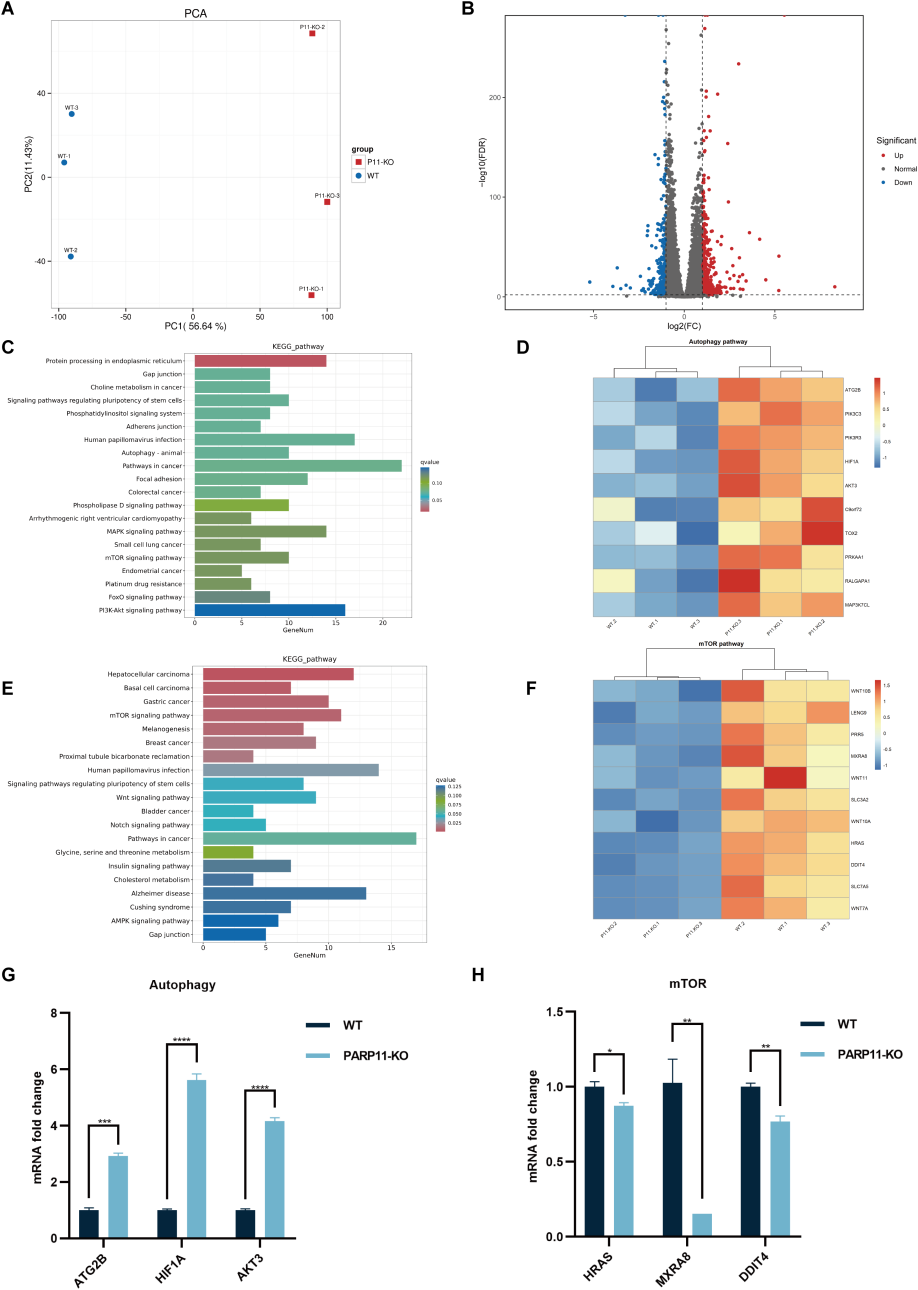
PARP11 knockout activated autophagy and interrupted mTOR pathway during PRV infection. **(A)** Principal component analysis (PCA) of replicates in each group. **(B)** The volcano plot of up- and down-regulated genes in PARP11-KO cells after PRV infection. **(C)** KEGG analysis of upregulated genes in PARP11-KO group. **(D)** The heatmap of enriched upregulated genes in autophagy pathway. **(E)** KEGG analysis of downregulated genes in PARP11-KO group. **(F)** The heatmap of enriched downregulated genes in mTOR pathway. **(G)** The expression of representative genes for the most enriched autophagy pathway. WT and PARP11-KO cells were infected with PRV (MOI = 0.01) for 12 h. ATG2B, HIF1A, and AKT3 were detected by RT-qPCR analysis. **(H)** The expression of representative genes for the most enriched mTOR pathway. WT and PARP11-KO cells were infected with PRV (MOI = 0.01) for 12 h. HRAS, MXRA8, and DDIT4 were detected by RT-qPCR analysis. Data are presented as means ± SE of three independent experiments, and asterisks (*) indicate the statistical significance: ns, no significance, *P < 0.05, **P < 0.01, ***P < 0.001, and ****P < 0.0001.

To further clear the host response against PRV in PARP11-KO cells, RNA sequencing was performed to compare the gene expression profiles of WT and PARP11-KO cells. Principal component analysis (PCA) was conducted to evaluate the reproducibility among the three replicates of each group. The results showed that the replicates at each group clustered together and separated clearly (**Figure 6A**). Differentially expressed genes (DEGs) were identified using an FDR < 0.05 and log2FC > 1.0 (upregulated genes) or log2FC < 1.0 (downregulated genes) as the cutoff criteria. Compared to the WT group, 820 DEGs were observed in the PARP11-KO group, in which 494 DEGs were upregulated and 326 DEGs were downregulated (**Figure 6B**). We conducted a linear fit analysis of RNA processing genes between RNA-seq and RT-qPCR changes. The result showed that the goodness of fit could reach 0.5150 (**Supplementary Figure S2C**).

Autophagy and mTOR pathway are essential components of host innate and adaptive immunity. Kyoto Encyclopedia of Genes and Genomes (KEGG) pathway analysis of above DEGs was performed to predict biological events elicited by PARP11-KO. We found that part of the upregulated DEGs was enriched in autophagy pathway (**Figures 6C, D**) and mTOR pathway was enriched among the downregulated DEGs (**Figures 6E, F**), which revealed that the autophagy pathway was activated and meanwhile the mTOR pathway was suppressed. Furthermore, the transcriptional level of autophagy related 2B (ATG2B), AKT serine/threonine kinase 3 (AKT3), and hypoxia inducible factor 1 subunit alpha (HIF1A) in autophagy pathway as well as HRas proto-oncogene (HRAS), matrix remodeling associated 8 (MXRA8), and DNA damage inducible transcript 4 (DDIT4) in mTOR pathway were confirmed by RT-qPCR. The result suggested that all three of these genes showed good agreement with RNA-seq data (**Figures 6G, H**).

**Figure 7:**
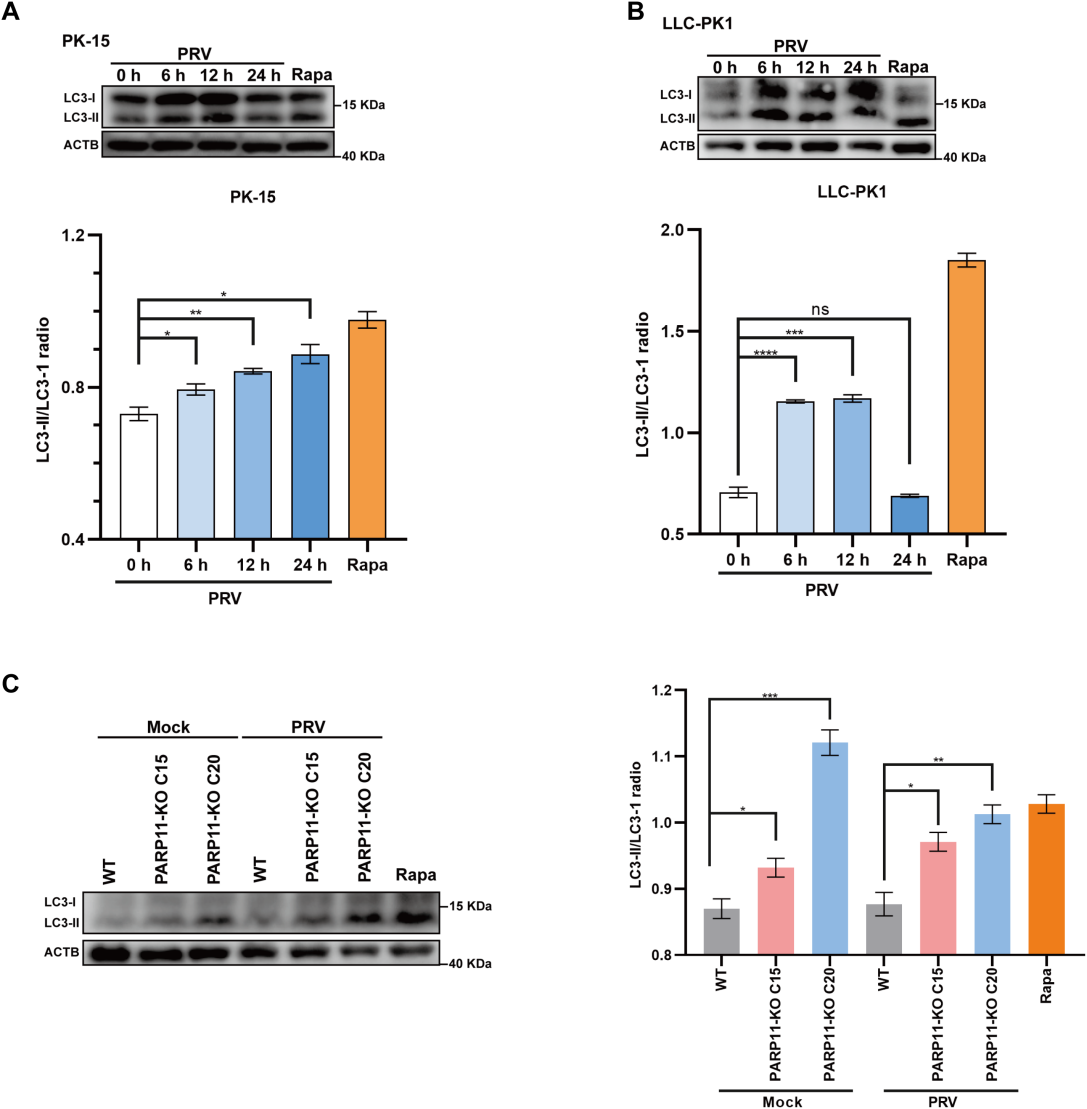
PARP11 knockout promoted LC3-I to LC3-II conversion. **(A)** Western blot analysis of LC3-II protein levels in PRV-infected PK-15 cells. **(B)** Western blot analysis of LC3-II protein levels in PRV-infected LLC-PK1 cells. **(C)** Western blot analysis of LC3-II protein levels in PARP11-KO cells. ACTB serves as a loading control and LC3-II abundance was quantified using Fiji software. Rapa, rapamycin, an autophagic inducer. Data are presented as means ± SE of three independent experiments, and asterisks (*) indicate the statistical significance: ns, no significance, *P < 0.05, **P < 0.01; ***P < 0.001, and ****P < 0.0001.

Previous studies have demonstrated that PRV infection induces autophagy (Wang et al., 2022; Xing et al., 2021b). To further elucidate the mechanism by which PARP11 restricts PRV infection, we first determined the expression level of LC3-II, an autophagic activation marker, in PRV-infected cells by western blot. As shown in **Figures 7A, B**, the ratio of LC3-II to LC3-I increased in both PRV-infected PK-15 and LLC-PK1 cells, suggesting that PRV infection significantly increased the endogenous LC3-I to LC3-II conversion. Next, the LC3-II level in PARP11-KO cells was also detected. As shown in **Figure 7C**, the ratio of LC3-II to LC3-I in PARP11-KO cells was higher than that in WT cells, indicating that PARP11-KO might promote autophagy pathway to further facilitate PRV infection. These data suggested that PARP11-KO promoted PRV infection via activating autophagy and suppressing mTOR pathway.

## 4 Discussion

PRV, a swine alphaherpesvirus closely related to the human herpes simplex virus 1 (HSV-1), has caused economic losses to the pig industry worldwide. In recent years, the cases that PRV cause human endophthalmitis and encephalitis were reported (Liu et al., 2021; Wong et al., 2019). Advancing the interplay between PRV and host will benefit swine industry and public health. A significant amount of research effort has been spent investigating host-virus interactions. In this study, we first confirmed that PRV infection inhibited the expression of PARP11. Then, we demonstrated that the inhibition of PARP11 induced by ITK7 and PARP11 knockout significantly promoted PRV replication. Further, we also found that PARP11 knockout can significantly facilitate mRNA export during PRV infection. Finally, RNA-seq indicated that PARP11 knockout suppressed PRV replication via activating autophagy pathway and suppressing mTOR pathway. These results suggested that PARP11 could be a novel antiviral target for the treatment of PRV infection.

PARP is a family of proteins with diverse functions including stress response, metabolism, viral infections. In this work, we identified PARP11 as a new host factor that was downregulated by PRV infection. Several PAPRs have been implicated in viral infections. For example, PARP9 KO in mouse was highly susceptible to infections with RNA viruses (e.g., VSV and reovirus) via impairing type I IFN production (Xing et al., 2021a). PARP1 inhibition assists E4orf4 in reducing adenovirus-induced DDR signaling and improves the efficiency of virus replication (Nebenzahl-Sharon et al., 2019). PARP12 can suppress Zika virus infection through degradation of NS1 and NS3 viral proteins (Li et al., 2018) and cooperation with PARP11 (Li et al., 2021b). Recently, it was reported that pharmacological and genetic inhibition of PARP1 significantly influenced PRV replication (Li et al., 2021a). However, how PARP11 is regulated by PRV and the role of PARP11 in PRV infection are still unknown. In this work, we first found that PRV infection could decrease PARP11 expression, and pharmacological and genetic inhibition of PARP11 significantly promoted PRV replication. Besides, the disturbance caused by PARP11-KO occurred in the transcription process of the immediate-early gene IE180, indicating PARP11 works on PRV replication at the early stage of viral infection.

PARP11 played diverse roles in different viral infection. Recently, it was suggested that PARP11 functioned as a host pro-virus factor to promote VSV infection through restricting IFN-I-induced antiviral efficacy. PARP11 promoted degradation of IFNAR1 via stabilizing ubiquitin E3 ligase β-TrCP (Guo et al., 2019). Another study demonstrated that PARP11 cooperated with PARP12 in restricting ZIKV infection through enhancing NS1 and NS3 degradation (Li et al., 2021b), indicating PARP11 was an anti-virus factor in ZIKV infection. In our study, we illustrated that PARP11 functioned as a restricted factor in PRV infection.

One of the common strategies utilized by viruses to promote viral transcription and replication was the acceleration of RNA export. For example, equine infectious anemia virus (EIAV) utilized the Rev-mediated CRM1 pathway to export incompletely spliced mRNA transcripts (Zhang et al., 2022). NXF1, a member of mRNA export pathway, was recruited by Ebola virus (EBOV) NP into cytoplasmic inclusion bodies to promote viral mRNA synthesis and translation (Wendt et al., 2020, 2022). Influenza A virus (IAV) NP interacted with NXT1 to promote viral replication (Chutiwitoonchai and Aida, 2016). To investigate whether the mRNA export pathway participated in the PRV increment caused by PARP11-KO, we detected the transcription of some factors (NXF1, NXT1, CRM1, and Nup98) involved in RNA export. As expected, these factors were universally upregulated in PRV-infected PARP11-KO cells. Recently, a report profiled the mono ADP-ribosylated (MARylated) targets of PARP11 *in vitro* using a chemical genetic strategy in HEK293T cells and indicated that the MARylated targets of PARP11 were involved in nuclear envelope organization and RNA transport (Carter-O’connell et al., 2016), which might support our results again. Additionally, the upstream of mRNA nuclear export was also enhanced in PARP11-KO cells, as speculated. Altogether, the activation of the RNA export and the RNA processing pathways in PARP11-KO cells may explain the phenotype of enhanced PRV replication.

Accumulating research investigating the interactions between PRV and host have focused on autophagy (Ming et al., 2021; Sun et al., 2017; Wang et al., 2022; Xing et al., 2021b; Xu et al., 2018). For instance, autophagy was induced in PK15 cells inoculated with PRV SX strain and autophagic inducer rapamycin-pretreated PK15 cells showed enhanced PRV SX replication (Xing et al., 2021b). Another study also suggested similar results in mouse neuro-2a cells and besides, this work demonstrated that autophagy inhibitor (3-MA) decreased the PRV ZJ01 progeny yields (Xu et al., 2018). However, these researched focused primarily on canonical autophagy factors such as ATG5, ATG7, and mTOR. In our work, we found PARP11-KO can activate autophagy pathway and suppress mTOR pathway during PRV infection via RNA-seq, indicating that PARP11 might play some roles in autophagy pathway. These results may not only explain the roles of PARP11 in PRV infection but also deepen our understanding the fundamental physiologic function.

In our study, we found that PRV infection could reduce PARP11 expression. Pharmacological and genetic inhibition of PARP11 can promote PRV replication. Mechanically, PARP11 restrict PRV replication via suppressing the mRNA export pathway hijacked by viruses. Furthermore, we suggested that PARP11 may play some roles in the autophagy pathway using the RNA-seq technique. In summary, we identified PARP11 as a host factor involved in PRV replication, expanding our understanding of PRV pathogenesis and providing a potential therapeutic target.

## Data Availability

The datasets presented in this study can be found in online repositories. The names of the repository/repositories and accession number(s) can be found below: https://www.ncbi.nlm.nih.gov/geo/query/acc.cgi?acc=GSE247973.
