# Supplementary material for: Identification of porcine PARP11 as a restricted factor for pseudorabies virus
Source: Front Cell Infect Microbiol. 2024 Oct 9;14:1414827. doi: 10.3389/fcimb.2024.1414827 (PMC11496260; doi:10.3389/fcimb.2024.1414827)
Supplement: Supplementary file 1 [file DataSheet1.docx]

Supplementary Material


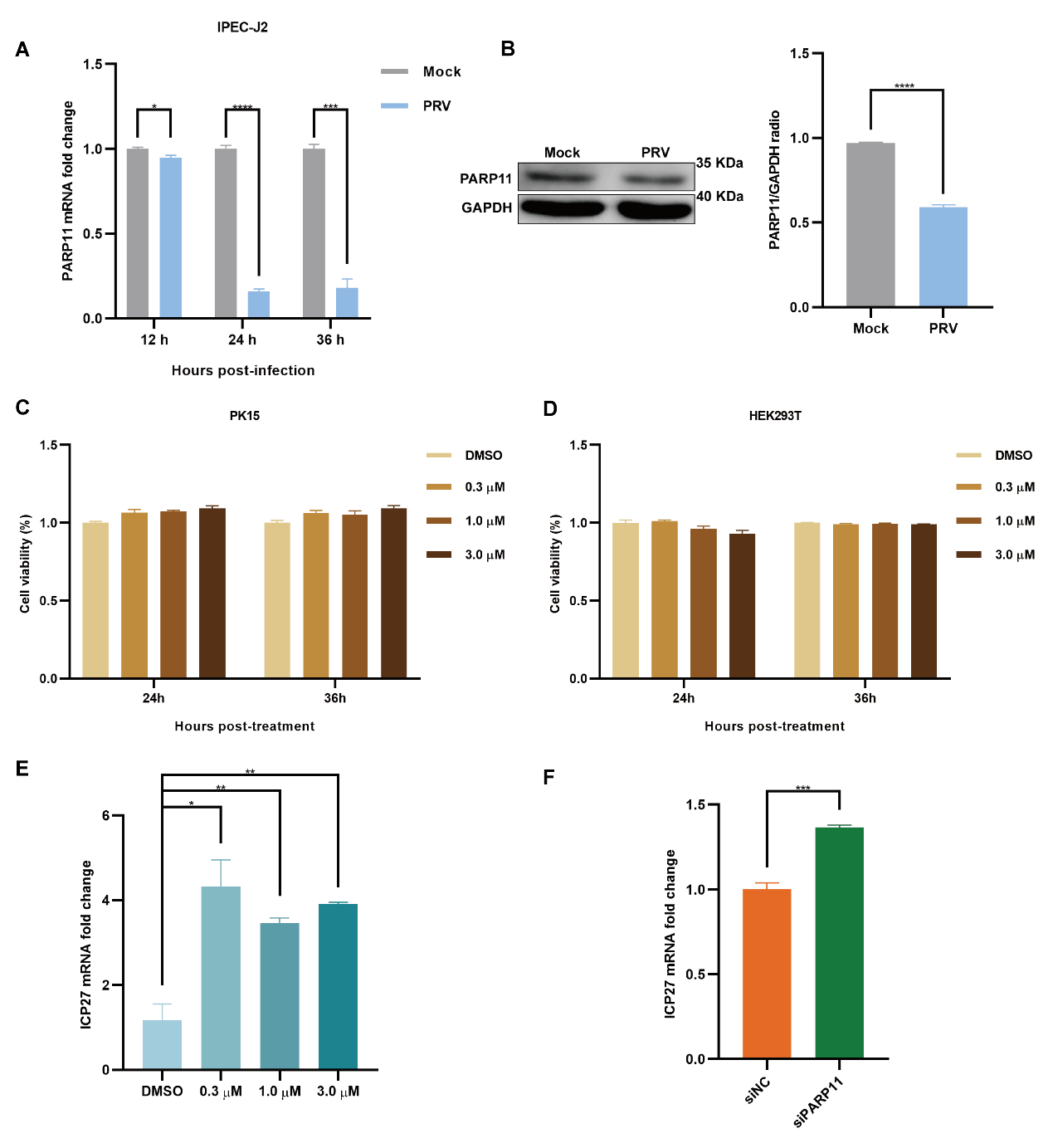


**Supplementary Figure 1.** The expression of PARP11 in IPEC-J2 cells following PRV infection and cell viability of PK15 and HEK293T treated with ITK7. (A) IPEC-J2 cells were infected with PRV (MOI = 0.1). PARP11 mRNA level was assessed by RT-qPCR analysis. (B) Western blot analysis of PARP11 protein levels in Marc145 cells infected with PRV (MOI = 0.01) for 24 h. GAPDH serves as a loading control and PARP11 abundance was quantified using Fiji software. Cell viability of PK15 (C) and HEK293T (D) cells treated with ITK7 (0.3 to 3.0 μM) by CCK-8 assays. (E) HEK293T cells were infected with PRV-BarthaK61 (MOI = 0.01) and simultaneously treated with ITK7 (0.3–3 μM) for 24 h. PRV ICP27 mRNA levels were assessed by RT-qPCR analysis. (F) PK15 cells were electro-transfected with siPARP11 for 12 h and then infected with PRV (MOI = 0.01) for 24 h. PRV ICP27 mRNA levels were assessed by RT-qPCR analysis.


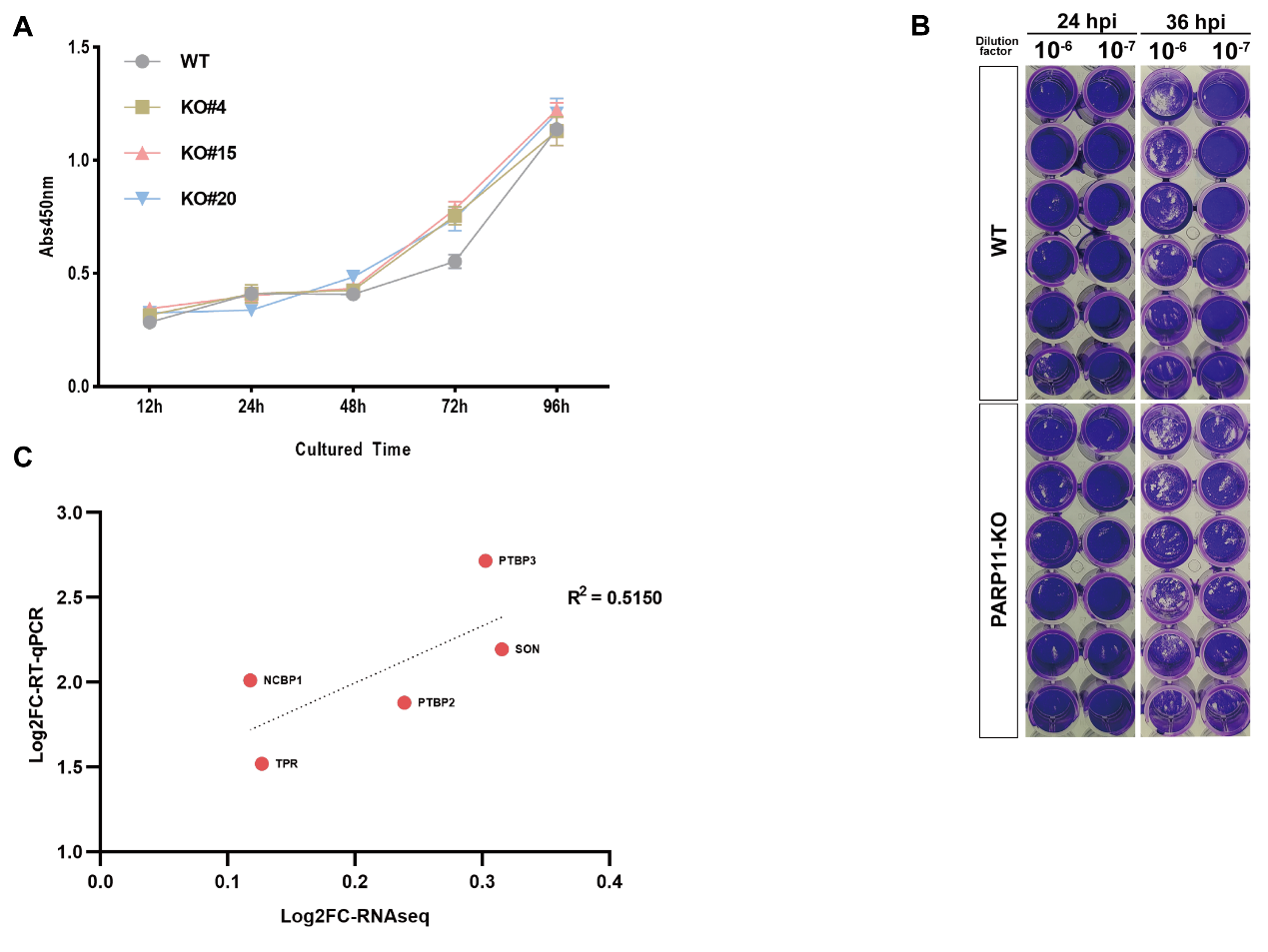


**Supplementary Figure 2.** The cell proliferation of PARP11-KO clones and PRV titers in PARP11-KO cells. (A) Cell viability of PARP11-KO cells. WT and PARP11-KO PK15 cells were cultured for 12-96 h and cell viability at indicated time was assessed by CCK-8 assay. (B) PRV titers in PARP11-KO cells. WT and PARP11-KO cells were infected with PRV-BarthaK61 (MOI = 0.01) for 24, 36 h. The supernatant was assessed by TCID50 assay. The cells were fixed at 72 hpi and plaques were visualized by staining with crystal violet. (C) The linear fit of RNA processing genes between RNA-seq and RT-qPCR changes.

**Supplementary Table 1. Primers and sequences in this research**

| Designation | Sequence (5’~3’) | Usage |
| --- | --- | --- |
| PARP11_F | ATGGGCACTAACATATCCTAC | PCR |
| PARP11_R | ACCTGATACATGTGCCACTT |  |
| PARP11_DL_F | TCTCACCACTGGAAAGCAGC | qPCR |
| PARP11_DL_R | TGATAAGCTGATAAGGCACCTCAG |  |
| sgPARP11_1F | CACCGCAAAACGACGGACAGCGAGA | crRNA |
| sgPARP11_1R | AAACTCTCGCTGTCCGTCGTTTTGC |  |
| sgPARP11_2F | CACCGAGAATTCTTAAACAAAACGA | crRNA |
| sgPARP11_2R | AAACTCGTTTTGTTTAAGAATTCTC |  |
| PRV_DL_F(gD) | GGTTCAACGAGGGCCAGTACCG | qPCR |
| PRV_DL_R(gD) | GCGTCAGGAATCGCATCACGT |  |
| CSFV_DL_F | CTAGCCATGCCCACAGTAGGA | qPCR |
| CSFV_DL_R | CTCCATGTGCCATGTACAGCA |  |
| ICP27_DL_F | TCACGTGGGTCAAGCTCATC | qPCR |
| ICP27_DL_R | ACCAGGGTGTAGGTGACGAT |  |
| IE180_DL_F | CATCGTGCTGGACACCATCGAG | qPCR |
| IE180_DL_R | ACGTAGACGTGGTAGTCCCCCA |  |
| EP0_DL_F | ACACCTCAGAGTCAGAGTGT | qPCR |
| EP0_DL_R | CTCGTCGATGCGCTGTTTGACATA |  |
| GAPDF_DL_F | GCCATCACCATCTTCCAGG | qPCR |
| GAPDH_DL_R | TCACGCCCATCACAAACAT |  |
| SON_DL_F | AATGAGTGAGCGGGCACTTG | qPCR |
| SON_DL_R | CCCGGTATTGGCCAACTGT |  |
| PTBP3_DL_F | CAGTCGGTTTAAAGCGGGGA | qPCR |
| PTBP3_DL_R | ACACACCTGTAGAAGGAGTGGA |  |
| TPR_DL_1F | TTGCTTCTCAAGGAGGTTTGGG | qPCR |
| TPR_DL_1R | AGTCACTGGGGCTGCTACTT |  |
| NCBP1_DL_F | ACAACGTGCGGCATGTCA | qPCR |
| NCBP1_DL_R | CCAAAGAGCAGGCACTCTTTTC |  |
| PTBP2_DL_F | GCTGTGACACCTCATCTTCGT | qPCR |
| PTBP2_DL_R | ACTGCTTGAGCACGTTGGT |  |
| NXF1_DL_F | GGAATGCCAGTCCCGATGAG | qPCR |
| NXF1_DL_R | TAGGCCACCAGGGAACATCC |  |
| NXT1_DL_F | ATGTTGCCTTCCAGCGAGTT | qPCR |
| NXT1_DL_R | GTTCTGGTTGAAGTCCCGCT |  |
| CRM1_DL_F | TCCGAGTTTGAGGCACTAGGA | qPCR |
| CRM1_DL_R | AGCAGCAAAGACTGGAACAGG |  |
| Nup98_DL_F | AAGACAATCCTGTGGAGGCG | qPCR |
| Nup98_DL_R | CTGCCAACCAGCTGGGATAA |  |
| ATG2B_DL_F | AGATCCTGGAGTCCGCAGAT | qPCR |
| ATG2B_DL_R | CAGAACCAGTTGCTAGGCGA |  |
| HIF1A_DL_F | TGCTGACCCTGCACTCAATC | qPCR |
| HIF1A_DL_R | CTGGGACTGTTAGGCTCAGGT |  |
| AKT3_DL_F | ATAATGACTATGGCCGAGCCG | qPCR |
| AKT3_DL_R | GTCCTCCACCAAGGCGTTT |  |
| HRAS_DL_F | ATCCACCAGTACAGGGAGCA | qPCR |
| HRAS_DL_R | GTCACACTTGTTCCCCACCA |  |
| MXRA8_DL_F | CCAAGTCAAAGGGGAAGGACG | qPCR |
| MXRA8_DL_R | TGGACTCACCTTTGTCCAAGTC |  |
| DDIT4_DL_F | AACTGCTCTAGCTGCGTCTTC | qPCR |
| DDIT4_DL_R | ACGAGAAGCGATCCCAAAGG |  |
